# Supplementary material for: Soil Mercury Pollution Changes Soil Arbuscular Mycorrhizal Fungal Community Composition
Source: J Fungi (Basel). 2023 Mar 23;9(4):395. doi: 10.3390/jof9040395 (PMC10143163; doi:10.3390/jof9040395)
Supplement: Supplementary file 1 [file jof-09-00395-s001.zip › jof-2286733-supplementary.pdf]

Article

Soil mercury pollution changes soil arbuscular mycorrhizal fungal community composition

Yidong Mi [1,2], Xue Bai [3], Xinru Li [2], Min Zhou [1,2], Xuesong Liu [2], Fanfan Wang [2], Hailei Su [3], Haiyan Chen [3] and Yuan Wei [2,*]

1 College of Environment, Hohai University, Nanjing 210098, China
2 State Key Laboratory of Environmental Criteria and Risk Assessment, Chinese Research Academy of Environmental Science, Beijing 100012, China
3 Department of Administration Service, Ministry of Ecology and Environment of the People's Republic of China, Beijing 100006, China
* Correspondence: craes_wy_team@163.com; Tel.: 010-84931804

Table S1 Location information for each sampling site.

Sampling site	Mining function	Latitude and longitude	Elevation (m)	Collected plants
S1	Tailing dam	109°14′57.60″ E, 27°31′47.19″ N	522	*A. argyi*, *E. indica*, *A. sinicus*, and *C. canadensis*
S2	Smelting plant	109°14′12.18″ E, 27°32′4.73″ N	607	*A. argyi*, *E. indica*, and *C. canadensis*
S3	Ore outlet	109°14′24.85″ E, 27°31′57.47″ N	574	*A. argyi*, *E. indica*, *A. sinicus*, and *C. canadensis*
S4	Tailing dam	109°11′36.45″ E, 27°33′35.41″ N	454	*A. argyi*, *E. indica*, *A. sinicus*, and *C. canadensis*
S5	Ore outlet	109°10′27.39″ E, 27°33′26.50″ N	444	*A. argyi*, *E. indica*, and *A. sinicu*
S6	Ore outlet	109°12′37.17″ E, 27°30′24.84″ N	848	*A. argyi*, *E. indica*, and *C. canadensis*
S7	Smelting plant	109°12′54.78″ E, 27°30′15.39″ N	856	*A. argyi*, *E. indica*, and *C. canadensis*

Table S2 Polymerase chain reaction (PCR) program and reaction system components

First round			
Reaction components		Volume/Mass	
5×FastPfu Buffer		4 μL	
2.5 mM deoxy-ribonucleoside triphosphates (dNTPs)		2 μL	
AML1		0.8 μL	
AML2		0.8 μL	
FastPfu polymerase		0.4 μL	
Template DNA		10 ng	
ddH_2O		20 μL	
Reaction parameters		Temperature (°C)	Time (s)
Initial denaturation		95	180
25 cycles	Denaturation	95	30
	Annealing	55	30
	Extension	72	45
Final extension		72	600
Refrigeration		10	
Second round			
Reaction components		Volume/Mass	
5×FastPfu Buffer		4 μL	
2.5 mM deoxy-ribonucleoside triphosphates (dNTPs)		2 μL	
AMV4.5NF		0.8 μL	
AMDGR		0.8 μL	
FastPfu polymerase		0.4 μL	
Template DNA		10 ng	
ddH_2O		20 μL	
Reaction parameters		Temperature (°C)	Time (s)
Initial denaturation		95	180
30 cycles	Denaturation	95	30
	Annealing	55	30
	Extension	72	45
Final extension		72	600
Refrigeration		10	

Table S3 Operational taxonomic units (OTUs) and sequence numbers of related species from each sampling site.

Family	Genus	Species	OTU	S1_AA	S1_EI	S1_AS	S1_CC	S2_AA	S2_EI	S2_CC	S3_AA	S3_EI
Archaeosporaceae	*Archaeospora*	*Archaeospora-Aca-*VTX00338	2	0	0	0	0	0	7	0	0	5
		*Archaeospora-trappei-*VTX00245	3	0	3	4	0	0	0	0	3019	0
Acaulosporaceae	*Acaulospora*	*Acaulospora-Acau10-*VTX00028	1	0	0	0	0	0	0	0	0	0
		*Acaulospora-Acau8-*VTX00027	2	0	0	0	0	1448	1757	455	0	0
		Unclassified *Acaulospora*	4	0	56	0	0	0	1290	0	0	0
Diversisporaceae	*Diversispora*	*Diversispora-MO-GC1-*VTX00060	1	0	0	1	0	0	2	0	0	0
		*Diversispora-sp.-*VTX00054	1	138	11	5	0	1978	2901	416	3879	1667
		Unclassified *Diversispora*	15	7573	10147	4044	2129	11016	12290	10352	17979	13344
	Diversisporaceae *Glomus*	*Glomus-versiforme-*VTX00061	4	5552	7722	11746	0	9152	2741	6828	2506	2770
	Unclassified Diversisporaceae	Unclassified Diversisporaceae	3	116	297	94	49	232	198	504	246	150
Gigasporaceae	*Scutellospora*	*Scutellospora-castanea-*VTX00041	1	0	0	0	0	0	0	0	0	0
Glomeraceae	Glomeraceae *Glomus*	*Glomus-acnaGlo2-*VTX00155	2	1280	206	11877	1735	0	0	0	0	0
		*Glomus-caledonium-*VTX00065	4	85	1	1	19554	3	0	0	5255	4637
		*Glomus-Douhan3-*VTX00212	1	22	239	2	0	357	0	1	0	0
		*Glomus-GlAd2.2-*VTX00210	1	0	0	0	0	0	0	0	0	0
		*Glomus-GlAd3.3-*VTX00289	1	0	0	0	0	0	0	0	0	0

	Glomus-Glo14-VTX00121	5	0	0	0	0	390	0	152	0	0
	Glomus-Glo16-VTX00120	6	0	0	0	0	298	0	402	0	0
	Glomus-Glo2-VTX00280	1	0	0	0	0	0	0	0	0	0
	Glomus-Glo3b-VTX00069	3	0	0	75	0	0	0	0	0	0
	Glomus-Glo7-VTX00214	1	79	108	315	135	0	0	0	0	0
	Glomus-Glo-C-VTX00323	1	0	0	0	0	0	0	0	0	0
	Glomus-group-B-Glomus-acnaGlo7-VTX00057	4	659	475	284	429	0	0	378	0	0
	Glomus-group-B-Glomus-Douhan9-VTX00056	1	0	0	0	0	0	0	0	0	0
	Glomus-group-B-Glomus-GlBb1.2-VTX00055	8	83	11936	1	57	11276	12113	20715	0	1
	Glomus-group-B-Glomus-Glo59-VTX00276	1	0	0	0	0	0	0	0	0	0
	Glomus-group-B-Glomus-Glo-G8-VTX00340	4	38	173	44	35	0	0	6	0	231
	Glomus-group-B-Glomus-lamellosu-VTX00193	9	5908	1952	7169	12287	64	178	3	4931	3610
	Glomus-group-B-Glomus-ORVIN-GLO4-VTX00278	3	0	0	0	0	254	149	0	0	0
	Glomus-group-B-Glomus-sp.-VTX00279	3	29	0	84	81	0	0	0	1	1
	Glomus-MO-G13-VTX00115	1	90	61	1	187	0	0	0	0	0
	Glomus-MO-G14-VTX00083	1	0	0	0	0	0	0	0	0	0
	Glomus-MO-G15-VTX00135	1	0	0	0	0	0	0	0	0	0
	Glomus-MO-G16-VTX00072	2	0	0	0	0	0	0	0	0	0
	Glomus-MO-G17-VTX00114	2	0	1	3	4696	0	0	0	2725	0

		Glomus-MO-G18-VTX00064	2	814	972	0	836	1189	464	1387	0	0
		Glomus-MO-G22- VTX00125	2	0	0	704	0	738	1609	550	0	135
		Glomus-MO-G23- VTX00222	3	0	0	4137	0	1812	1106	1219	0	366
		Glomus-mosseae- VTX00067	2	108	7548	0	1647	2223	1	0	6666	19054
		Glomus-ORVIN-GLO3E- VTX00309	1	0	0	335	0	0	0	0	0	0
		Glomus-perpusillum- VTX00287	1	0	0	0	0	94	0	0	0	0
		Glomus-sp.-VTX00165	1	31	298	296	0	0	0	0	1	0
		Glomus-sp.-VTX00301	1	0	0	0	0	0	0	0	0	0
		Glomus-sp.-VTX00304	2	179	1	0	0	0	0	0	1150	176
		Glomus-sp.-VTX00330	2	823	0	0	22	433	101	148	0	0
		Glomus-viscosum- VTX00063	9	584	391	294	0	286	266	0	2071	2422
		Glomus-Wirsel-OTU12- VTX00188	2	0	0	412	0	0	0	0	0	0
		Glomus-Wirsel-OTU14- VTX00137	1	0	0	0	0	0	0	0	0	0
		Glomus-Wirsel-OTU16- VTX00156	2	578	204	659	0	0	0	177	1216	1156
		Glomus-Wirsel-OTU6- VTX00202	3	0	0	0	0	0	0	0	0	0
		Glomus-Yamato08-A1- VTX00100	1	0	0	0	0	0	0	0	0	0
		Unclassified *Glomeraceae Glomus*	77	27712	9290	7847	8657	6183	11550	8205	621	2901
Paraglomeraceae	*Paraglomus*	*Paraglomus-brasilianum-* VTX00239	1	0	0	0	0	0	171	0	0	0

		*Paraglomus-Glom-1B.13-*VTX00308	6	12	0	56	15	0	0	0	0	0
		*Paraglomus-Para1-OTU2-*VTX00337	1	0	0	203	0	236	228	0	0	0
		*Paraglomus-Para1-*VTX00336	1	0	0	0	0	0	0	0	0	0
		Paraglomus-sp.-VTX00001	1	0	0	2	0	0	0	0	0	0
		Unclassified *Paraglomus*	3	24	510	2	49	2525	3297	431	0	0
Unclassified Glomeromycetes	Unclassified Glomeromycetes	Unclassified Glomeromycetes	88	125	40	1945	42	455	223	313	376	16
Family	Genus	Species	S3_AS	S3_CC	S4_AA	S4_EI	S4_AS	S4_CC	S5_AA	S5_EI	S5_AS	S6_AA
Archaeosporaceae	*Archaeospora*	*Archaeospora-Aca-*VTX00338	0	0	0	0	10	0	0	15	0	0
		*Archaeospora-trappei-*VTX00245	0	0	0	0	0	0	0	1	1	0
Acaulosporaceae	*Acaulospora*	*Acaulospora-Acau10-*VTX00028	505	0	0	0	0	0	0	0	0	0
		*Acaulospora-Acau8-*VTX00027	793	0	3875	0	0	0	0	0	1	0
		Unclassified *Acaulospora*	1	0	3718	0	0	0	42	9606	2259	5
Diversisporaceae	*Diversispora*	*Diversispora-MO-GC1-*VTX00060	0	0	0	0	0	0	0	0	0	796
		*Diversispora-sp.-*VTX00054	1391	1231	77	6	20	1	0	15331	3708	35
		Unclassified *Diversispora*	7728	9933	6524	10226	5482	1	454	8972	11271	3637
	Diversisporaceae *Glomus*	*Glomus-versiforme-*VTX00061	10127	18840	5083	608	20906	699	68	9	56	0
	Unclassified Diversisporaceae	Unclassified Diversisporaceae	308	348	98	94	306	0	21	272	92	223
Gigasporaceae	*Scutellospora*	*Scutellospora-castanea-*VTX00041	0	0	0	0	0	0	0	0	1	0

Glomeraceae	Glomeraceae *Glomus*	*Glomus-acnaGlo2*-VTX00155	0	0	0	0	0	0	0	0	14	0
		Glomus-caledonium-VTX00065	1849	2450	156	3606	0	0	372	2008	647	1
		Glomus-Douhan3-VTX00212	0	0	1	0	0	2	0	1122	11	108
		Glomus-GlAd2.2-VTX00210	0	0	0	0	0	0	0	0	0	0
		Glomus-GlAd3.3-VTX00289	0	0	0	0	0	0	0	0	0	0
		Glomus-Glo14-VTX00121	0	0	1	2084	0	107	0	1	0	0
		Glomus-Glo16-VTX00120	0	0	656	2432	480	3099	2	0	0	1
		Glomus-Glo2-VTX00280	0	0	0	0	0	0	0	0	0	0
		Glomus-Glo3b-VTX00069	0	0	95	214	0	0	0	0	0	33
		Glomus-Glo7-VTX00214	0	0	296	0	185	101	9	0	0	1246
		Glomus-Glo-C-VTX00323	0	0	288	0	0	0	0	0	0	0
		Glomus-group-B-Glomus-acnaGlo7-VTX00057	0	592	579	140	174	0	82	626	853	16
		Glomus-group-B-Glomus-Douhan9-VTX00056	0	0	0	0	0	0	0	0	1	0
		Glomus-group-B-Glomus-GlBb1.2-VTX00055	1	0	1060	56	6991	3687	24078	0	5709	10131
		Glomus-group-B-Glomus-Glo59-VTX00276	0	0	0	0	0	2	0	0	0	0
		Glomus-group-B-Glomus-Glo-G8-VTX00340	0	0	78	122	0	0	1	585	38	3
		Glomus-group-B-Glomus-lamellosu-VTX00193	20628	7482	572	2480	5173	3228	5029	4938	842	650
		Glomus-group-B-Glomus-ORVIN-GLO4-VTX00278	0	0	0	73	164	1416	0	179	19	0
		Glomus-group-B-Glomus-sp.-VTX00279	252	133	0	0	4	1	0	0	0	0

	*Glomus-MO-G13-*VTX00115	0	0	0	113	0	0	11	0	0	596
	*Glomus-MO-G14-*VTX00083	0	0	394	0	0	0	0	0	0	0
	*Glomus-MO-G15-*VTX00135	0	0	104	265	0	564	0	0	0	70
	*Glomus-MO-G16-*VTX00072	0	0	979	0	0	313	0	0	0	0
	*Glomus-MO-G17-*VTX00114	1139	1534	0	0	0	2	38	0	0	1
	Glomus-MO-G18-VTX00064	0	0	5705	87	0	0	15	1	1	1163
	*Glomus-MO-G22-*VTX00125	145	0	604	58	0	46	0	0	0	47
	*Glomus-MO-G23-*VTX00222	1941	0	240	284	443	407	0	0	93	141
	*Glomus-mosseae-*VTX00067	487	1091	6285	2901	1	250	19428	1829	185	126
	*Glomus-ORVIN-GLO3E-*VTX00309	0	0	0	0	0	0	0	0	0	0
	*Glomus-perpusillum-*VTX00287	0	0	0	0	0	0	0	0	0	52
	Glomus-sp.-VTX00165	0	0	0	0	0	0	0	0	0	647
	Glomus-sp.-VTX00301	0	0	0	0	0	0	0	0	0	0
	Glomus-sp.-VTX00304	350	0	138	0	151	0	0	0	0	0
	Glomus-sp.-VTX00330	0	326	0	0	0	1	45	0	0	0
	*Glomus-viscosum-*VTX00063	2276	2002	813	716	1118	298	121	4271	335	201
	*Glomus-Wirsel-OTU12-*VTX00188	0	0	0	106	0	17	45	1	0	75
	*Glomus-Wirsel-OTU14-*VTX00137	0	0	543	77	0	86	25	0	0	0

		*Glomus-Wirsel-OTU16-*VTX00156	426	0	218	0	214	0	1	617	0	0
		*Glomus-Wirsel-OTU6-*VTX00202	0	0	1071	476	851	4206	86	0	0	0
		*Glomus-Yamato08-A1-*VTX00100	0	0	0	0	0	430	0	0	0	0
		Unclassified *Glomeraceae Glomus*	2007	6625	12303	23159	9810	25684	2009	2257	25763	32409
Paraglomeraceae	*Paraglomus*	*Paraglomus-brasilianum-*VTX00239	0	0	0	0	17	0	0	0	0	0
		*Paraglomus-Glom-1B.13-*VTX00308	0	0	0	0	0	0	0	0	0	0
		*Paraglomus-Para1-OTU2-*VTX00337	0	0	69	1617	0	1814	0	1	446	172
		*Paraglomus-Para1-*VTX00336	0	0	7	0	0	0	0	0	0	0
		Paraglomus-sp.-VTX00001	0	0	0	0	0	0	0	0	0	0
		Unclassified *Paraglomus*	0	0	1	603	0	6158	660	0	214	3
Unclassified Glomeromycetes	Unclassified Glomeromycetes	Unclassified Glomeromycetes	288	55	11	39	142	22	0	0	82	54

Family	Genus	Species	S6_EI	S6_CC	S7_AA	S7_EI	S7_CC	S0_AA
Archaeosporaceae	*Archaeospora*	*Archaeospora-Aca-*VTX00338	0	0	32	25	6	0
		*Archaeospora-trappei-*VTX00245	2	0	0	0	0	0
Acaulosporaceae	*Acaulospora*	*Acaulospora-Acau10-*VTX00028	0	0	0	0	0	0
		*Acaulospora-Acau8-*VTX00027	0	0	0	0	0	0
		Unclassified *Acaulospora*	0	0	146	2	0	0
Diversisporaceae	*Diversispora*	*Diversispora-MO-GC1-*VTX00060	1	0	0	0	0	0

		Diversispora-sp.-VTX00054	5079	936	18	16	8	4
		Unclassified *Diversispora*	3486	8311	2254	6688	4521	18245
	Diversisporaceae *Glomus*	*Glomus-versiforme*-VTX00061	988	100	18367	10043	4249	923
	Unclassified Diversisporaceae	Unclassified Diversisporaceae	85	53	203	291	207	244
Gigasporaceae	*Scutellospora*	*Scutellospora-castanea*-VTX00041	0	0	0	0	0	510
Glomeraceae	Glomeraceae *Glomus*	*Glomus-acnaGlo2*-VTX00155	0	0	1	0	0	0
		Glomus-caledonium-VTX00065	11383	1	1	98	380	9101
		Glomus-Douhan3-VTX00212	459	820	0	0	0	0
		Glomus-GlAd2.2-VTX00210	0	0	101	85	0	0
		Glomus-GlAd3.3-VTX00289	0	0	816	79	0	0
		Glomus-Glo14-VTX00121	0	138	589	2	0	0
		Glomus-Glo16-VTX00120	115	3253	7	143	0	1
		Glomus-Glo2-VTX00280	0	0	0	0	0	107
		Glomus-Glo3b-VTX00069	48	127	0	21	0	0
		Glomus-Glo7-VTX00214	1227	351	1	0	85	0
		Glomus-Glo-C-VTX00323	0	0	0	0	0	0
		Glomus-group-B-Glomus-acnaGlo7-VTX00057	1216	603	728	1495	143	221
		Glomus-group-B-Glomus-Douhan9-VTX00056	0	284	0	48	0	0
		Glomus-group-B-Glomus-GlBb1.2-VTX00055	5661	2239	897	385	9175	2

	Glomus-group-B-Glomus-Glo59-VTX00276	2	373	936	0	3	0
	Glomus-group-B-Glomus-Glo-G8-VTX00340	61	1	6	24	4	0
	Glomus-group-B-Glomus-lamellosu-VTX00193	5151	2809	3676	11764	10094	5177
	Glomus-group-B-Glomus-ORVIN-GLO4-VTX00278	129	0	1005	282	0	0
	Glomus-group-B-Glomus-sp.-VTX00279	19	0	2	0	3	1
	Glomus-MO-G13-VTX00115	89	97	3	364	68	0
	Glomus-MO-G14-VTX00083	124	0	229	0	0	0
	Glomus-MO-G15-VTX00135	0	0	79	31	0	0
	Glomus-MO-G16-VTX00072	0	0	0	0	0	0
	Glomus-MO-G17-VTX00114	0	0	421	123	288	0
	Glomus-MO-G18-VTX00064	1113	82	1190	219	374	1
	Glomus-MO-G22-VTX00125	0	0	0	0	0	464
	Glomus-MO-G23-VTX00222	248	0	0	0	0	1205
	Glomus-mosseae-VTX00067	748	249	777	3058	16724	2718
	Glomus-ORVIN-GLO3E-VTX00309	0	0	0	0	0	0
	Glomus-perpusillum-VTX00287	0	0	1057	0	115	0
	Glomus-sp.-VTX00165	0	0	0	68	0	112

		Glomus-sp.-VTX00301	173	0	75	0	0	0
		Glomus-sp.-VTX00304	60	161	243	142	64	281
		Glomus-sp.-VTX00330	0	262	0	0	0	440
		*Glomus-viscosum-*VTX00063	251	1271	2073	411	1554	4478
		*Glomus-Wirsel-OTU12-*VTX00188	15	0	140	135	1	0
		*Glomus-Wirsel-OTU14-*VTX00137	1	543	98	91	0	162
		*Glomus-Wirsel-OTU16-*VTX00156	455	277	370	327	268	238
		*Glomus-Wirsel-OTU6-*VTX00202	0	0	261	456	160	0
		*Glomus-Yamato08-A1-*VTX00100	0	0	124	0	88	119
		Unclassified *Glomeraceae Glomus*	14016	28638	14821	14558	4058	7065
Paraglomeraceae	*Paraglomus*	*Paraglomus-brasilianum-*VTX00239	0	0	421	363	0	0
		*Paraglomus-Glom-1B.13-*VTX00308	0	0	0	0	0	0
		*Paraglomus-Para1-OTU2-*VTX00337	223	0	424	192	0	110
		*Paraglomus-Para1-*VTX00336	0	0	0	0	0	0
		Paraglomus-sp.-VTX00001	0	0	0	0	0	0
		Unclassified *Paraglomus*	1	519	0	603	0	89
Unclassified Glomeromycetes	Unclassified Glomeromycetes	Unclassified Glomeromycetes	13	144	50	10	2	624

Table S4 Permutational multivariate ANOVA (PERMANOVA) showing the Bray–Curtis distance-based dissimilarity of arbuscular mycorrhizal fungal (AMF) communities among the different sampling sites.

Group	Df	SS	MS	F Model	R^2	Pr (>F)
Sampling sites	6	2.400	0.400	1.852	0.395	0.001
Plant	3	0.677	0.226	0.837	0.112	0.760

Note: SS: sum of squares; Df: degree of freedom; MS: mean square.

Table S5 Correlation coefficients and R-squared and significance values (*p* value) for canonical correspondence analysis (CCA) axes. (Fig. 3).

	CCA1	CCA2	R^2	*p* value
PC1	0.869	-0.495	0.617	0.001
PC2	-0.468	0.884	0.054	0.558
PC3	0.554	0.833	0.609	0.001

Table S6 Results of the general linear mixed models on Sobs, Shannon, and Shannoneven index of AMF communities.

		Estimate	SE	Df	*t* value	*p* value	AIC
Sobs	Intercept	82.000	3.356	20	24.431	0.000	183.338
	PC1	2.836	3.429	20	0.827	0.418	
	PC2	2.045	3.429	20	0.596	0.558	
	PC3	–9.915	3.429	20	–2.892	0.009	
Shannon	Intercept	2.635	0.078	20	33.761	0.000	32.891
	PC1	–0.062	0.080	20	–0.777	0.446	
	PC2	–0.115	0.080	20	–1.446	0.164	
	PC3	–0.321	0.080	20	–4.026	0.001	
Shannoneven	Intercept	0.599	0.014	20	42.785	0.000	-35.845
	PC1	–0.019	0.014	20	–1.359	0.189	
	PC2	–0.031	0.014	20	–2.190	0.041	
	PC3	–0.055	0.014	20	–3.868	0.001	

Note: SE: standard error; Df: degree of freedom; AIC: akaike information criterion.

Table S7 Pearson correlations between soil properties and arbuscular mycorrhizal fungi (AMF) richness and diversity using canonical correspondence analysis.

Soil properties	Sobs	Shannon	Shannoneven
AK	0.579(0.003)**	0.234(0.270)	0.077(0.719)
AP	–0.088(0.681)	–0.414(0.044)*	–0.485(0.016)*
AN	0.094(0.663)	–0.051(0.811)	–0.094(0.662)
TK	–0.150(0.485)	–0.364(0.081)	–0.394(0.057)
TP	0.030(0.891)	–0.373(0.073)	–0.486(0.016)*
TN	0.117(0.587)	0.015(0.944)	–0.025(0.909)
THg	–0.424(0.039)*	–0.347(0.097)	–0.263(0.214)
TOC	0.174(0.415)	–0.141(0.511)	–0.234(0.271)
pH	0.173(0.418)	0.010(0.964)	–0.058(0.787)
WC	0.400(0.053)	0.490(0.015)*	0.466(0.022)*

Significance levels: *, $p < 0.05$; **, $p < 0.01$; ***, $p < 0.001$. THg: total mercury; AK: available potassium; AP: available phosphorus; AN: available nitrogen; TK: total potassium; TP: total phosphorus; TN: total nitrogen; TOC: total organic carbon; WC: water content.

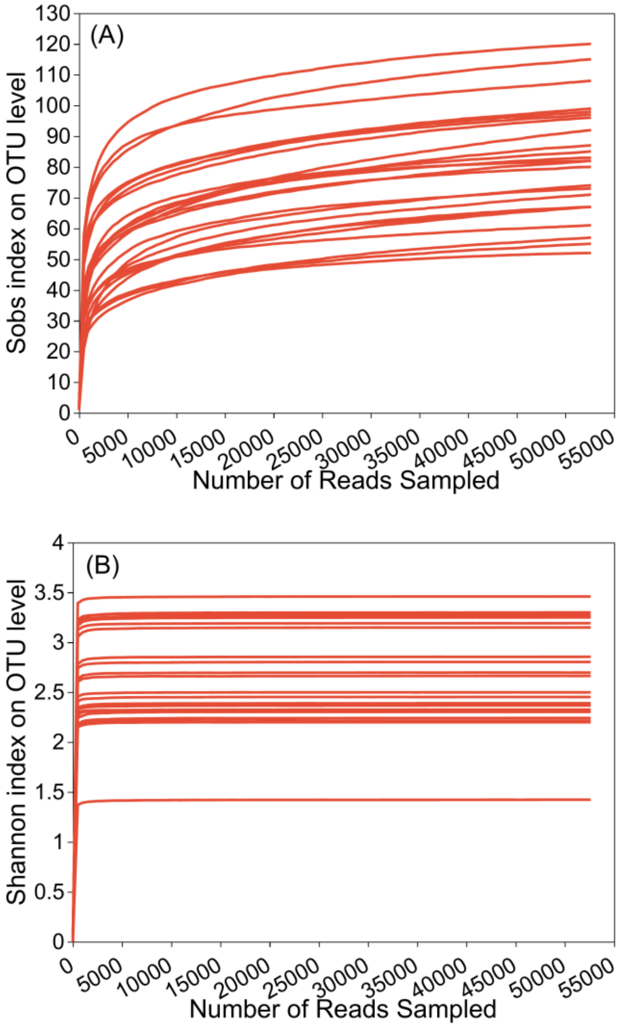
(A)
Sobs index on OTU level
Number of Reads Sampled
0
10
20
30
40
50
60
70
80
90
100
110
120
130
0
5000
10000
15000
20000
25000
30000
35000
40000
45000
50000
55000
(B)
Shannon index on OTU level
Number of Reads Sampled
0
0.5
1
1.5
2
2.5
3
3.5
4

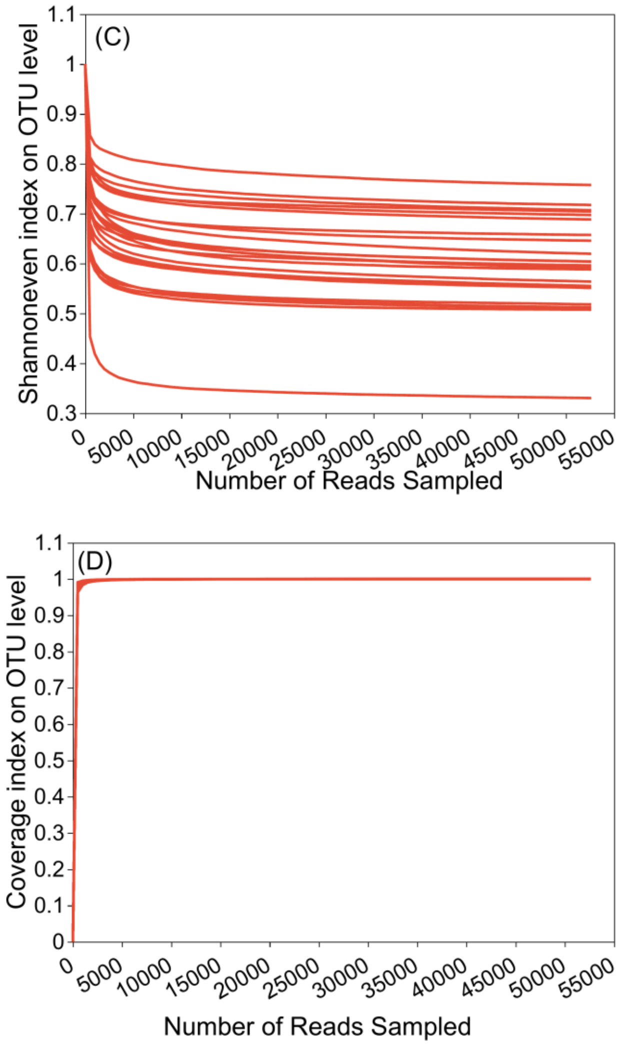

Figure S1 Rarefaction analysis for observed species (Sobs) (A), Shannon (B), Shannoneven (C), and Coverage (D) indexes of arbuscular mycorrhizal fungi based on sample reads numbers.

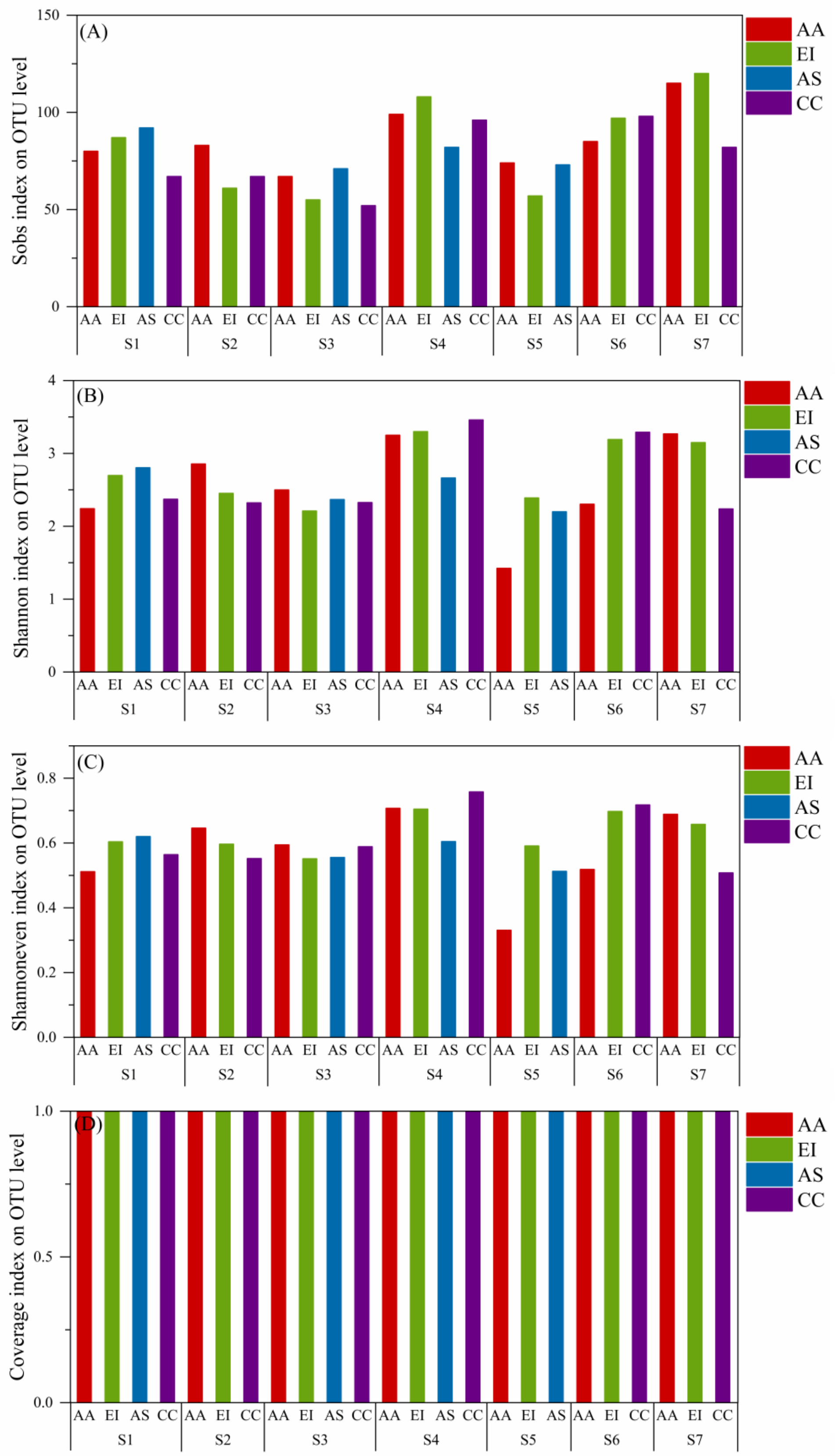

Figure S2 Richness, diversity, evenness, and coverage estimates for communities of arbuscular mycorrhizal fungi using the Sobs (A), Shannon (B), Shannoneven (C), and Coverage (D) indexes at all sampling sites. Aa: *Artemisia argyi*, Ei: *Eleusine indica*, As: *Astragalus sinicus*, and Cc: *Conyza canadensis*.

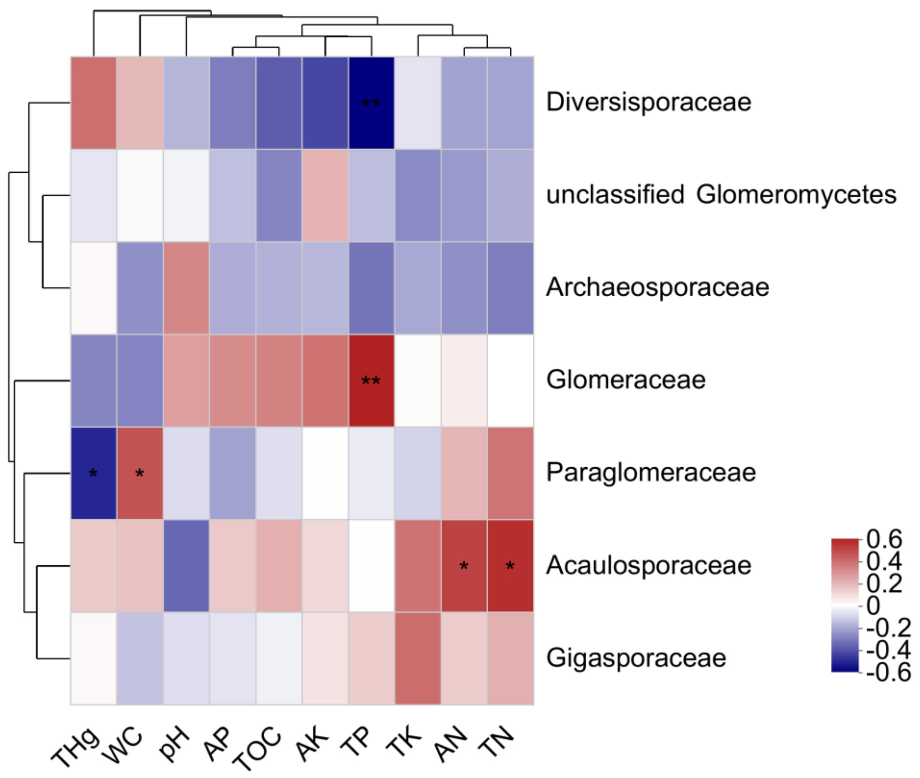

Figure S3 Community heatmap analysis based on the Pearson correlation coefficients of soil properties and the AMF richness on the family level. Variations in correlation coefficients are shown by color gradients, as indicated by the scale to the right. The different colors on the left represent different families. *: $p < 0.05$; **: $p < 0.01$. THg: total mercury; AK: available potassium; AP: available phosphorus; AN: available nitrogen; TK: total potassium; TP: total phosphorus; TN: total nitrogen; TOC: total organic carbon; WC: water content.
